# Nanostructure Effect on Magnetization Processes in FePt Polytwin Crystals

**DOI:** 10.3390/ma16216863

**Published:** 2023-10-26

**Authors:** Jingwei Yi, Xinyu Xu, Wenqin Yue, Feiyang Liu, Jingzhi Hu, Liwei D. Geng

**Affiliations:** Department of Materials Science and Engineering, Sichuan University-Pittsburgh Institute, Sichuan University, Chengdu 610065, China

**Keywords:** polytwin boundary, phase-field modeling, magnetization process, domain mechanism

## Abstract

In FePt polytwin crystals with large magnetocrystalline anisotropy, the boundaries may play a crucial role in the magnetization processes occurring under an external magnetic field. In this study, we employed phase-field modeling and computer simulations to systematically investigate the effect of three types of polytwin boundaries—namely, symmetric (Type I), asymmetric (Type II), and mixed (Type III) boundaries—on magnetization processes as well as coercive fields under an external magnetic field along various directions. Because of the large anisotropy of FePt, the domain wall motion mechanism is usually dominant in the domain switching processes, while the magnetization rotation mechanism only becomes important at the late magnetization stage under a high external magnetic field. Among these three types of polytwin boundaries, the low coercivity is mainly due to the domain wall motion process, which starts from the intersection point at the polytwin boundary. The coercive field for the mixed polytwin boundary (Type III) is always in between the values of Type I and II.

## 1. Introduction

The ordered L1_0_ phase of FePt demonstrates exceptional magnetic anisotropy energy, rendering it a promising candidate for high-density magnetic recording media and high-energy product materials in microelectromechanical systems [1,2,3]. With a tetragonal lattice and magnetic easy direction along the tetragonal c-axis, the ordered L1_0_ phase originates from the cubic disordered A1 phase, giving rise to three crystal variants with tetragonal axes oriented along the <100> directions, each comprising two antiphase domains. The ordering reaction induces elastic energy due to lattice misfit between different crystal variants during the FePt system’s ordering process. This energy can be effectively mitigated through the formation of stress-accommodating crystallographic microstructures [4]. Among the typical microstructures observed in bulk FePt, multilayers composed of alternating {110} twin-related crystallographic variants, referred to as polytwin microstructures, have garnered significant attention due to the eliminated misfit stress.

**Figure 1:**
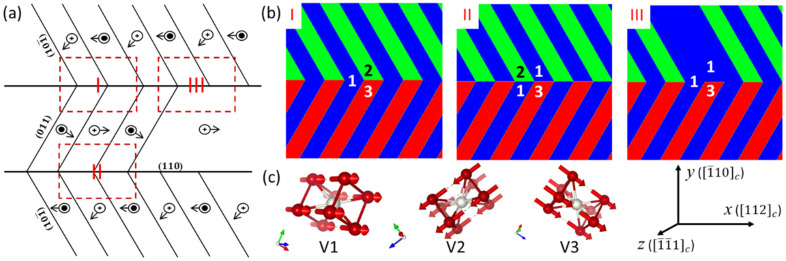
(**a**) Schematic illustration of the typical FePt polytwin microstructure with {110} twin boundaries and antiphase domains. The vertical lines represent polytwin boundaries. (**b**) Phase morphologies of three types of polytwin boundaries: symmetric (Type I), asymmetric (Type II), and mixed (Type III). The three crystal variants are represented by the blue, green, and red colors, respectively. (**c**) Atomic orientations of the three crystal variants: V1, V2, and V3. The red arrows represent the spin direction of each domain variant. The global coordinate system is located in the bottom right of the figure.

The typical FePt polytwin microstructure is schematically illustrated in Figure 1a, which is reproduced from relevant experimental observations [5,6,7]. The structure comprises three crystal variants with tetragonal axes that are oriented along the <100> directions, denoted as V1, V2, and V3 in Figure 1c. Figure 1a also depicts the {110} twin boundaries and antiphase domains resulting from the three crystal variants. In addition to the twin boundaries and antiphase domains, the polytwin boundaries, represented by the vertical lines in Figure 1a, also exist in this polytwin microstructure. According to the grain orientation and crystal symmetry, the polytwin boundaries can be classified into three types: symmetric (Type I), asymmetric (Type II), and mixed (Type III). The phase morphologies of these three types are illustrated in Figure 1b.

Extensive investigations have been conducted on FePt polytwin crystals to comprehend the correlation between the microstructures and magnetic properties. Particularly, the {110} twin boundaries and antiphase domains in FePt polytwin crystals have been subjected to experimental and theoretical studies concerning their influence on magnetic domain structures and magnetic properties [5,6,7,8,9,10,11,12,13,14]. However, the effect of polytwin boundaries on magnetic domain structures and properties has rarely been investigated. Although the microstructures and magnetic domain structures of polytwin boundaries were experimentally observed using Lorentz microscopy [5,6,7], the respective magnetic properties that were influenced by different polytwin boundary types were quite difficult to examine in the experiments. Nevertheless, computer simulations have been one of the best method to explore the nanostructure effect on magnetic properties. Prior simulations have only focused on magnetization processes associated with twin boundaries rather than polytwin boundaries [8]. In this paper, we employ phase-field modeling and computer simulations to conduct a systematic study into the effects of three types of polytwin boundaries on magnetization processes. Basically, magnetic constants such as exchange stiffness, magnetocrystalline anisotropy, and saturation magnetization at the boundary will differ from those of bulk, and even the surface magnetic anisotropy will appear at the boundaries. In this work, we consider boundaries that are sharp enough to make those differences negligible.

## 2. Phase-Field Modeling

To simulate the magnetization evolution process in FePt polytwin microstructures with different polytwin boundary types, we employ the micromagnetic phase-field model [15]. The polytwin crystal structure of the L1_0_ phase is characterized by a spatially distributed vector field t(r), which describes the local orientation of the tetragonal axis; the three crystal variants are depicted in Figure 1c. Additionally, the magnetic domain structure is represented by another vector field M(r), which describes the magnetization distribution. Because the magnitude of magnetization is the constant $M_{s}$, the time-evolving unit vector field $m\left(r\right)=M\left(r\right)/M_{s}$ is often used to describe the magnetic domain structure and its evolution. The evolution of the magnetization is governed by the Landau–Lifshitz–Gilbert equation [15,16]:

(1) $$\dot{m}=\gamma H^{\mathrm{eff}}\times m+\alpha m\times \dot{m},$$

where $\dot{m}$ is the time derivative of **m**, γ is the gyromagnetic ratio, and α is the damping parameter. The effective magnetic field $H^{\mathrm{eff}}$ is the variational derivative of the total free energy with respect to **M**:

(2) $$H^{\mathrm{eff}}=-1/\mu_{0}\left(\delta F/\delta M\right),$$

where $\mu_{0}$ denotes the vacuum permeability. The total free energy of the FePt polytwin system consists of the magnetocrystalline anisotropy energy, exchange energy, magnetostatic energy, and external magnetic energy (Zeeman energy), which can be formulated as:

(3) F=∫fan(m(r))d3r+A∫|grad[m(r)]|2d3r+μ02∫d3k(2π)3|n⋅M˜(k)|2−μ0∫Hext⋅M(r)d3r

where *K*_1_ represents the magnetocrystalline anisotropy constant, *A* is the exchange stiffness constant, and **H**^ext^ is the external magnetic field. Equation (1) is solved numerically through the utilization of our own Fortran 90 code, which employs a parallel algorithm facilitated by the message passing interface (MPI). This code was executed on the supercomputers at the Hefei Advanced Computing Center, which was previously employed in our other studies [17,18]. The material parameters of FePt adopted in this study are [8]: *M*_s_ = 1.14 × 10^6^ A/m, *K*_1_ = 6.6 × 10^6^ J/m^3^, and *A* = 10^−11^ J/m. The computational cell of 512 × 512 × 1 with periodic boundary condition is used to describe the magnetization evolution in FePt polytwin crystals. The computational grid size is 1.4 nm, and the magnetic domain size is about 78 nm.

## 3. Results and Discussion

**Figure 2:**
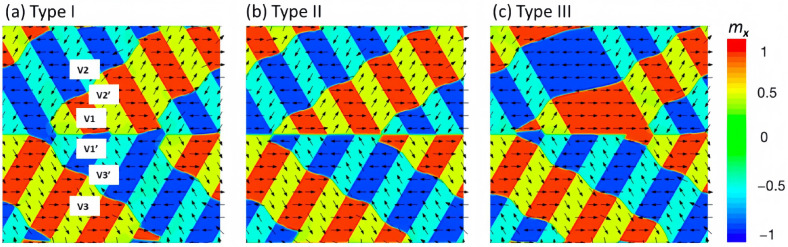
Simulated initial magnetic domain structures of FePt polytwin crystals with three types of polytwin boundaries: (**a**) symmetric (Type I), (**b**) asymmetric (Type II), and (**c**) mixed (Type III). The six magnetic domain variants are denoted as V1, V1′, V2, V2′, V3, and V3′. Black vectors represent the in-plane magnetization components *m_x_* and *m_y_*, while the color contours the component *m_x_*.

**Figure 3:**
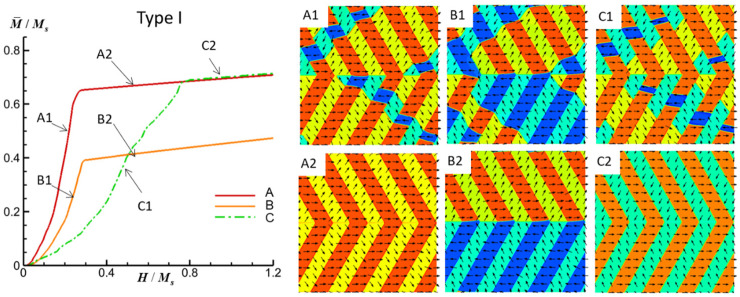
Simulated M-H curves and magnetic domain structures of an FePt polytwin crystal with a Type I (symmetric) polytwin boundary under an external magnetic field along the *x*-axis (A), *y*-axis (B), and *z*-axis (C). For magnetic domain structures, black vectors represent the in-plane magnetization components *m_x_* and *m_y_*, while the color contours the component *m_x_*. The initial magnetic domain structure is shown in Figure 2a.

**Figure 4:**
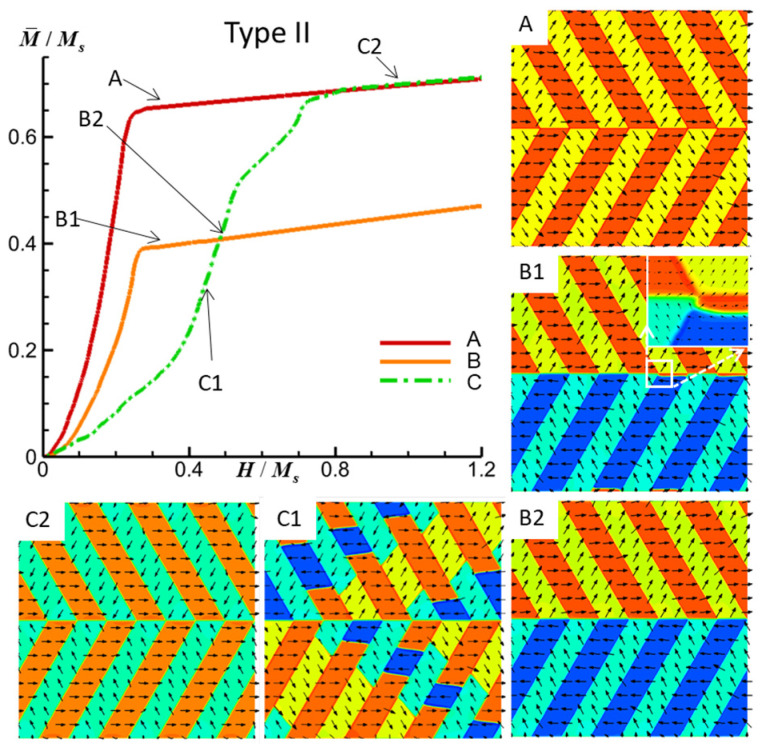
Simulated M-H curves and magnetic domain structures of an FePt polytwin crystal with a Type II (asymmetric) polytwin boundary under an external magnetic field along the *x*-axis (A), *y*-axis (B), and *z*-axis (C). For magnetic domain structures, black vectors represent the in-plane magnetization components *m_x_* and *m_y_*, while the color contours the component *m_x_*. The initial magnetic domain structure is shown in Figure 2b.

**Figure 5:**
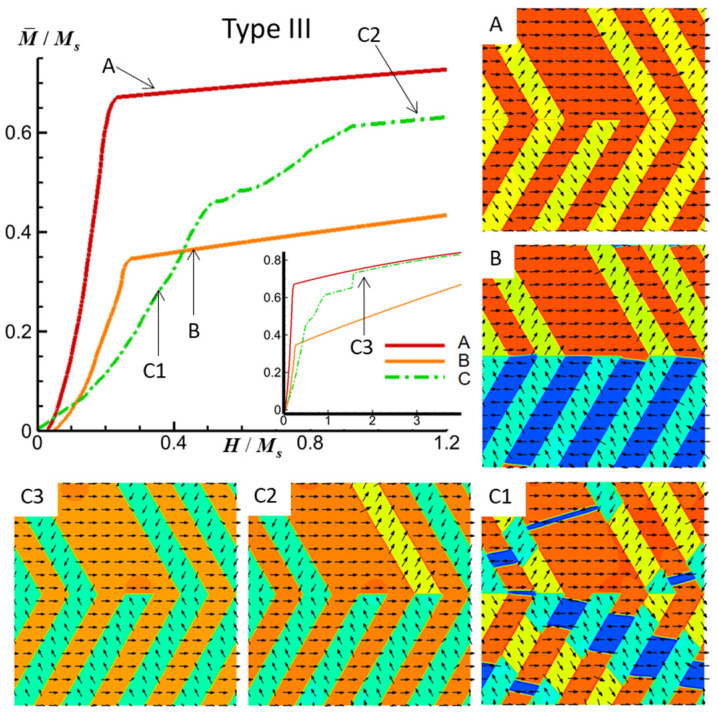
Simulated M-H curves and magnetic domain structures of an FePt polytwin crystal with a Type III (mixed) polytwin boundary under an external magnetic field along the *x*-axis (A), *y*-axis (B), and *z*-axis (C). For magnetic domain structures, black vectors represent the in-plane magnetization components *m_x_* and *m_y_*, while the color contours the component *m_x_*. The initial magnetic domain structure is shown in Figure 2c.

**Figure 6:**
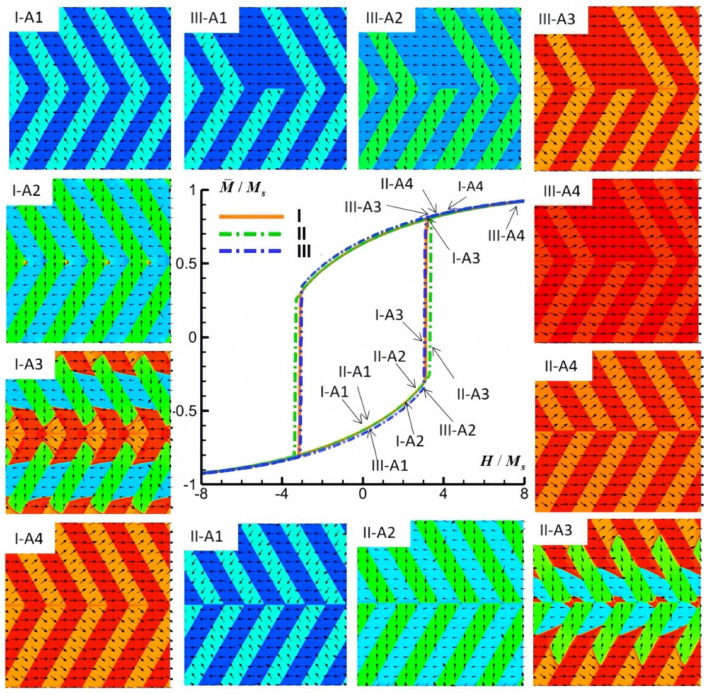
Simulated M-H hysteresis curves and magnetic domain structures of an FePt polytwin crystal with Type I (symmetric), Type II (asymmetric), and Type III (mixed) polytwin boundaries under an external magnetic field along the *x*-axis (A). For magnetic domain structures, black vectors represent the in-plane magnetization components *m_x_* and *m_y_*, while the color contours the component *m_x_*.

**Figure 7:**
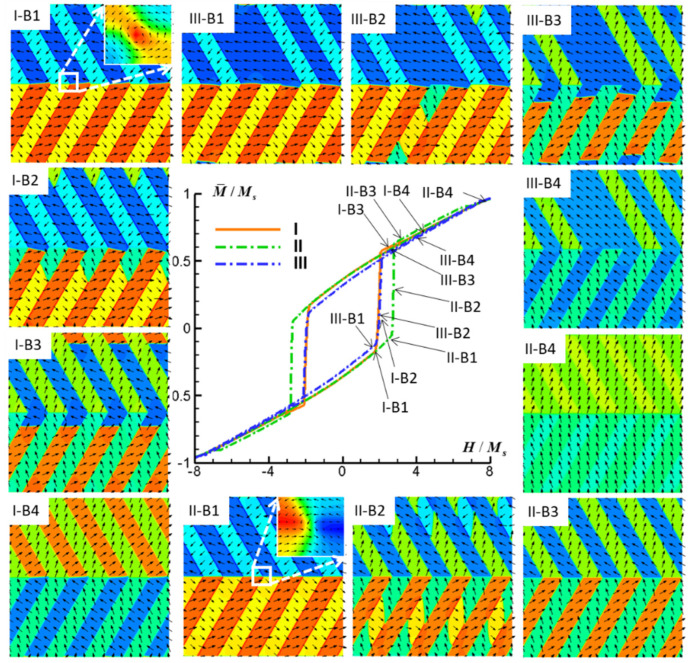
Simulated M-H hysteresis curves and magnetic domain structures of an FePt polytwin crystal with Type I (symmetric), Type II (asymmetric), and Type III (mixed) polytwin boundaries under an external magnetic field along the *y*-axis (B). For magnetic domain structures, black vectors represent the in-plane magnetization components *m_x_* and *m_y_*, while the color contours the component *m_x_*. The color of the insets in I-B1 and II-B1 contours the out-of-plane component *m_z_*.

**Figure 8:**
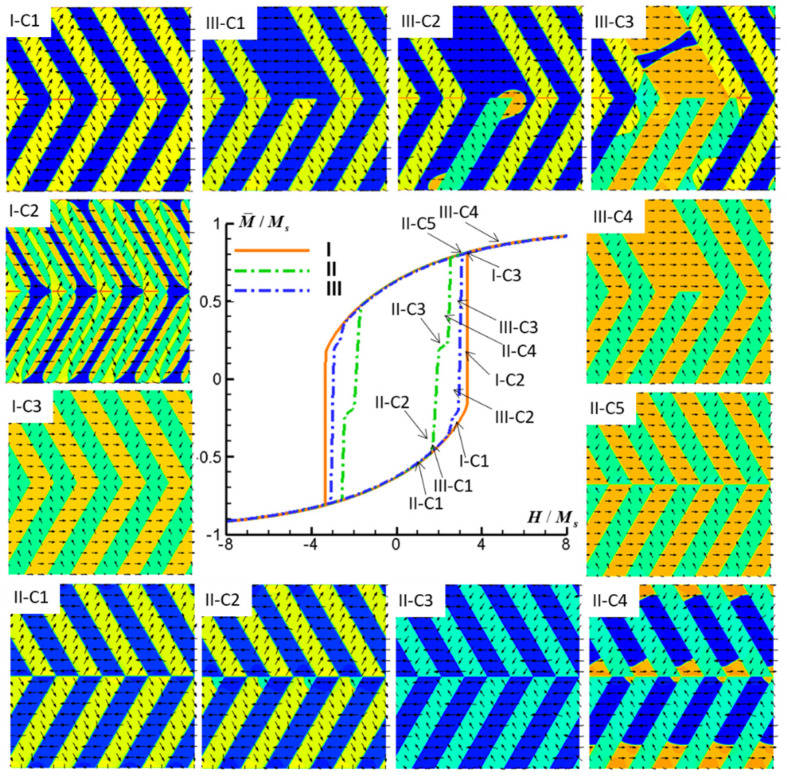
Simulated M-H hysteresis curves and magnetic domain structures of an FePt polytwin crystal with Type I (symmetric), Type II (asymmetric), and Type III (mixed) polytwin boundaries under an external magnetic field along the *z*-axis (C). For magnetic domain structures, black vectors represent the in-plane magnetization components *m_x_* and *m_y_*, while the color contours the component *m_x_*.

To investigate the influence of polytwin boundaries on magnetization processes, we consider three distinct FePt polytwin systems; their corresponding phase morphologies are visually depicted in Figure 1b. The simulations start by initializing the magnetic domain structures within the FePt polytwin crystals, incorporating three types of polytwin boundaries: symmetric (Type I), asymmetric (Type II), and mixed (Type III), as delineated in Figure 2 . The strong tetragonal magnetocrystalline anisotropy results in the alignment of all magnetization vectors along their respective underlying tetragonal c-axes. Within this context, the polytwin crystal encompasses three crystal variants, each characterized by two antiphase domains, thereby yielding six distinct domain variants denoted as V1, V1′, V2, V2′, V3, and V3′. This configuration is presented in Figure 2a. Black vectors represent the magnetization components *m_x_* and *m_y_*, while the color contours the component *m_x_*. Figure 3, Figure 4, Figure 5, Figure 6, Figure 7 and Figure 8 use the same color scale as in Figure 2. The antiphase domains within each crystal variant are separated by domain walls. Notably, these internal domain walls are 180 degrees, forming parallel orientations with the c-axes. For instance, the V1-V1′ domain wall aligns parallelly with the polytwin boundary, while the V2-V2′ or V3-V3′ domain walls deviate by 60 degrees from the polytwin boundary or the *x*-axis. Consequently, the domain walls connected to neighboring domain walls manifest as a zigzag arrangement that is characterized by an average deviation angle of 30 degrees from the polytwin boundary.

However, the influence of exchange coupling between adjacent domain walls introduces deviations from the ideal 180-degree configuration. These quasi-180-degree domain walls observed in Figure 2 display a slightly smoothed zigzag pattern while still maintaining the consistent average deviation angle of 30 degrees from the polytwin boundary. Importantly, these quasi-180-degree domain walls exhibit higher energy levels compared with their ideal 180-degree counterparts, which is primarily attributable to the accumulation of magnetic charges along the walls.

The domain structures within FePt polytwin crystals, as exemplified in Figure 2a–c, reveal similarities across the considered cases. A detailed inspection of these domain structures, however, highlights a discernible dissimilarity that is primarily localized at the polytwin boundaries. In the context of the symmetric polytwin boundary configuration, a distinction arises through the presence of two distinct domain wall types. Specifically, the V1-V1′ quasi-180-degree domain wall and the V2-V3 or V2′-V3′ 60-degree domain wall, both depicted in Figure 2a, play pivotal roles. The former experiences a slight deviation from alignment with the polytwin boundary, while the latter assumes parallel alignment with the boundary. Transitioning to the asymmetric polytwin boundary depicted in Figure 2b, the presence of a misfit between V1-V2 or V1-V3 crystal variants introduces elevated energy levels at the boundary compared with Type I configurations. Furthermore, the pronounced charge accumulation along the domain walls at this boundary also contributes to its increased energy relative to Type I boundaries. For the mixed polytwin boundary (Type III), a coexistence of magnetically charged and charge-free domain walls is observed, as detailed in Figure 2c.

In the pursuit of comprehending the magnetization evolution processes within FePt polytwin crystals, we turn our attention to the influence of three distinct types of polytwin boundaries in the presence of an external magnetic field. The investigation encompasses three representative field orientations, specifically the field being aligned with the *x*-axis (Case A), *y*-axis (Case B), and *z*-axis (Case C). Figure 3 illustrates the simulated M-H (magnetization field) curves and corresponding magnetic domain structure evolutions for the FePt polytwin crystal featuring Type I (symmetric) polytwin boundaries under external magnetic fields aligned in different directions.

Beginning with the application of a magnetic field along the *x*-direction, a notable evolution is observed in the movement of the three distinct antiphase domain walls. Consequently, the domains V1, V2′, and V3 undergo continuous growth, while the domains V1′, V2, and V3′ diminish and eventually vanish, as illustrated in Figure 3(A1,A2). The resultant state reveals the existence of solely the domains V1, V2′, and V3. Notably, at the polytwin boundary, the initial V1-V1′ quasi-180-degree domain wall transitions, making way for the emergence of a highly charged V2′-V3 120-degree domain wall, which replaces the initial charge-free V2-V3 or V2′-V3′ 60-degree domain wall.

Upon the application of a magnetic field along the *y*-direction, distinct behavior becomes apparent. Unlike the scenario in Case A, Case B reveals the continuous growth of the V2′ and V3′ domains accompanied by the diminishment and eventual vanishing of the V2 and V3 domains, as demonstrated in Figure 3(B1,B2). The evolution of the V1 and V1′ domains without the *m_y_* component is influenced by their neighboring domains. Consequently, the final state manifests the presence of solely the V1 and V2′ domains in the upper section, while the lower section retains the V1′ and V3′ domains. The polytwin boundary in this scenario features two types of domain walls, namely the V1–V1′ quasi-180-degree domain walls and the V2′–V3′ 60-degree domain walls, both of which are characterized by an absence of magnetic charges.

Lastly, the application of a magnetic field along the *z*-direction induces the continuous growth of the V1, V2, and V3′ domains, which is coupled with the diminishing and eventual disappearance of the remaining domains. This evolution corresponds to the motion of the antiphase domain walls, as depicted in Figure 3(C1,C2). At the polytwin boundary, the initial V1-V1′ quasi-180-degree domain wall is replaced by a highly charged V2-V3′ 120-degree domain wall, thus supplanting the initial charge-free V2-V3 or V2′-V3′ 60-degree domain wall. Additionally, twin boundaries featuring the head-to-head domain configurations of V1-V2 and V1-V3′ are also found to be highly charged, contributing to the overall understanding of the magnetic landscape within this polytwin crystal system.

Figure 4 illustrates the simulated M-H curves and the associated magnetic domain structure evolutions within the FePt polytwin crystal with Type II (asymmetric) polytwin boundaries. The external magnetic field is oriented in three directions for a comprehensive analysis. The initial configuration of magnetic domains is depicted in Figure 2b. Given that the structural disparity between Type I and Type II configurations solely resides in the nature of the polytwin boundary, it follows that any discernible differences in domain structures must be attributed to the polytwin boundary region. Upon subjecting the system to a magnetic field along the *x*-direction, the domains V1, V2′, and V3 undergo progressive growth while the remaining domains diminish until their eventual disappearance, as shown in Figure 4A. At the polytwin boundary, the presence of two distinct highly charged domain walls is notable: the V1-V2′ 60-degree domain wall and the V1-V3 60-degree domain wall.

Subsequently, when a magnetic field is applied along the *y*-direction, a distinct pattern emerges. Continuous growth is observed for the V2′ and V3′ domains, which is accompanied by the diminishing of the V2 and V3 domains. Consequently, the V1 domains expand within the upper region, whereas the V1′ domains proliferate in the lower section. Figure 4(B1) presents the diminutive V1 domains in the lower portion; a tiny V1 domain is enlarged in the inset, which will be eventually eliminated at the polytwin boundary. The final state reveals the persistence of solely the V1 and V2′ domains in the upper section, which coexist with the presence of V1′ and V3′ domains in the lower portion, as demonstrated in Figure 4(B2). Notably, the polytwin boundary in this configuration hosts two distinct types of highly charged domain walls: the V1-V3′ 60-degree domain wall and the V1′-V2′ 60-degree domain wall. Lastly, the application of a magnetic field along the z-direction results in the continuous growth of the V1, V2, and V3′ domains, accompanied by the eventual vanishing of the remaining domains, as depicted in Figure 4(C1,C2). Notably, at the polytwin boundary, two distinct types of highly charged domain walls are evident: the V1-V3′ 60-degree domain wall and the V1-V2 60-degree domain wall.

Figure 5 presents the simulated M-H curves alongside the corresponding magnetic domain structure evolutions within the FePt polytwin crystal exhibiting Type III (mixed) polytwin boundaries under an external magnetic field along three directions. The initial magnetic domain configuration is depicted in Figure 2c. Notably, the structural characteristics of Type III closely resemble those of Type I, as the substitution of a single V2 crystal variant from the upper portion of Type I with V1 constitutes the primary distinction. As a result, the magnetic domain structures of Type III bear marked resemblance to those of Type I.

Figure 5A,B illustrate the replacement of one V2′ domain within the upper region with the V1 domain. Further examination under a magnetic field in the *z*-axis, as displayed in Figure 5(C1–C3), provides insight into the domain evolution process. As the system reaches the final state, it is observed that one of the V2 domains within the upper region undergoes substitution with the V1 domain, substantiating the earlier structural observations and underscoring the correspondence between Type III and Type I configurations.

The simulated M-H hysteresis curves and concomitant magnetic domain structure evolutions within the FePt polytwin crystal, characterized by three distinct categories of polytwin boundaries (I, II, III) and subjected to external magnetic fields aligned along the *x*-axis, *y*-axis, and *z*-axis, are systematically depicted in Figure 6, Figure 7 and Figure 8.

Figure 6 presents a comprehensive illustration of the simulated M-H hysteresis curves and magnetic domain structures exhibited by the FePt polytwin crystal for all three polytwin boundary types when subjected to an external magnetic field aligned along the *x*-axis. In the context of magnetic domain switching mechanisms, two pivotal processes typically contribute to the phenomenon: magnetization rotation and domain wall motion. Notably, domain wall motion often transpires more readily than magnetization rotation for facilitating domain switching. Figure 6(I-A1–I-A4) systematically portray the intricate domain evolution process associated with the symmetric polytwin boundary configuration. Upon the imposition of a magnetic field along the *x*-direction, magnetizations align themselves with the field. As the field strength attains sufficient magnitude, the switching mechanism originates at the intersection point of the polytwin boundary. This pivotal event triggers the nucleation of the V2′ and V3 domains alongside the concurrent generation of V2-V2′ and V3-V3′ antiphase domain walls that encircle the intersection point. Consequently, the transition process primarily unfolds through domain wall motion, which supersedes the role of magnetization rotation. The newly formed domain walls swiftly propagate in response to the magnetic field, leading to a distinct and sharp amplification in the hysteresis curve. Following the eventual dissipation of V2 and V3′ domains, the ongoing augmentation in magnetization is primarily attributed to the magnetization rotation process.

In the case of an FePt polytwin crystal endowed with an asymmetric Type II boundary, a similar sequence of events emerges. After the field-driven magnetization rotation, the switching process initiates at the intersection point of the polytwin boundary, which is followed by the cascading domain wall motion emanating from the vicinity of this intersection point. This mechanism is expounded in Figure 6(II-A1–II-A4). Importantly, both configurations exhibit analogous domain switching behaviors and concomitant M–H hysteresis loops, underscoring the inherent similarities in their respective magnetic response characteristics. Notably, the coercive field of Type I is slightly lower than that of Type II. Figure 6(III-A1–III-A4) present a comprehensive depiction of the domain evolution process that is intrinsic to the mixed polytwin boundary scenario (Type III). As anticipated, the application of an external magnetic field incites the switching process at the intersection point of the polytwin boundary, which is succeeded by the progressive mobilization of antiphase domain walls. The resultant M-H hysteresis curve manifests close alignment with that of the Type I configuration, substantiating the anticipated similarities in their magnetic response behaviors.

Figure 7 presents a comprehensive depiction of the simulated M-H hysteresis curves and the corresponding magnetic domain structures observed within the FePt polytwin crystal for all three distinct polytwin boundaries when subjected to an external magnetic field along the *y*-axis. Figure 7(I-B1–I-B4) systematically elucidate the intricate domain evolution process inherent to the symmetric polytwin boundary (Type I). Upon subjecting the crystal to a magnetic field along the *y*-direction, the switching mechanism originates at the intersection point of the polytwin boundary. This initiation leads to the formation of V2′ and V3′ domains accompanied by the consequent emergence of V2-V2′ and V3-V3′ antiphase domain walls encircling the intersection point, as illustrated in Figure 7I-B2. Notably, these nascent domain walls propagate with rapidity under the influence of the magnetic field, culminating in a distinct and pronounced escalation in the hysteresis curve. Subsequent to the annihilation of V2 and V3 domains, the twin boundaries undergo a transition into a highly charged state. Consequently, the motion of V1-V1′ domain walls, marked by sluggish propagation as depicted in Figure 7I-B3, contributes marginally to the hysteresis curve. Indeed, the augmentation in magnetization witnessed subsequent to the swift switching in the hysteresis curve primarily arises from magnetization rotation.

Figure 7(II-B1–II-B4) present a comprehensive illustration of the domain evolution process intrinsic to the asymmetric polytwin boundary (Type II). Analogous to the symmetric counterpart, the switching mechanism originates at the intersection point of the polytwin boundary and is followed by a rapid progression of antiphase domain walls, resulting in a swift switching process. The Type I polytwin crystal manifests a domain structure that is characterized by a vortex configuration at the intersection point as shown in the inset of Figure 7I-B1, typically exhibiting heightened mobility under external fields. This enhanced mobility contributes to the ease of switching and, consequently, results in a lower coercivity compared with that of Type II. It is worth noting that such a vortex configuration does not exist in Type II, as shown in the inset of Figure 7I-B1. Lastly, Figure 7(III-B1–III-B4) comprehensively outline the domain evolution process inherent to the mixed polytwin boundary scenario (Type III). As foreseen, the hysteresis curve and corresponding domain structure evolution closely resemble the characteristics observed in the Type I configuration.

Figure 8 presents the simulated M-H hysteresis curves and concurrent evolution of magnetic domain structures within the FePt polytwin crystal characterized by the three distinct polytwin boundary configurations when subjected to an external magnetic field aligned along the *z*-axis. Figure 8(I-C1–I-C3) provide a comprehensive portrayal of the domain evolution process associated with the symmetric polytwin boundary scenario (Type I) under the influence of a magnetic field along the *z*-axis. contrast to the above scenarios discussed earlier, the domain switching mechanism in this case occurs at the twin boundaries rather than at the polytwin boundary. In these twin boundary regions, the rapid nucleation and growth of V1, V2, and V3′ domains transpire, ultimately leading to their complete occupation of the polytwin crystal.

Figure 8(II-C1–II-C5) present a detailed depiction of the domain evolution process intrinsic to the asymmetric polytwin boundary configuration (Type II) when subjected to the *z*-axis magnetic field. In contrast to Type I configurations, the switching process takes place at the intersection point of the polytwin boundary, which is followed by subsequent antiphase domain wall motion. Notably, this process involves the rapid nucleation and growth of V3′ domains from the intersection point accompanied by swift V3-V3′ domain wall motion, resulting in the initial pronounced escalation of magnetization. Following this, V1 domains nucleate from the same intersection point, exhibiting rapid V1-V1′ domain wall motion; this leads to a second sharp increase in magnetization and the appearance of a kink in the M-H curve. Due to the fact that the switching mechanism transpires at the polytwin boundary for Type I crystals, these crystals exhibit a larger coercivity compared with that of Type II. Lastly, Figure 8(III-C1–III-C4) comprehensively outline the domain evolution process intrinsic to the mixed polytwin boundary configuration (Type III) under the influence of a *z*-axis magnetic field. As anticipated, the domain switching process commences in the Type II region, wherein domains nucleate from the intersection point of the polytwin boundary. Consequently, the coercivity observed in this configuration is lower than that of Type I.

**Figure 9:**
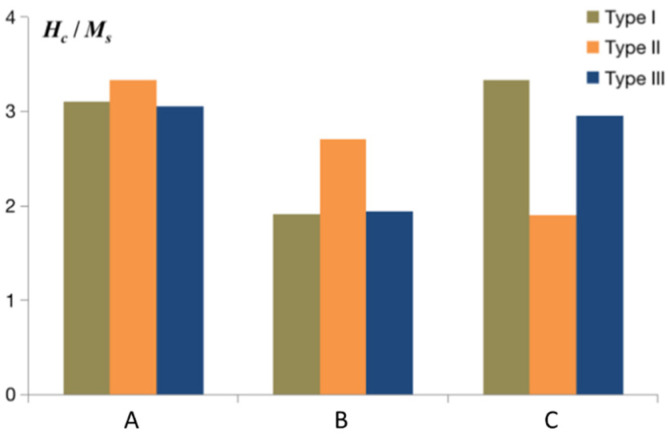
Simulated magnetic coercive field of FePt polytwin crystal with Type I (symmetric), Type II (asymmetric), and Type III (mixed) polytwin boundaries under an external magnetic field along the *x*-axis (A), *y*-axis (B), and *z*-axis (C).

The magnetic coercive field pertaining to the FePt polytwin crystals, which encompasses all three polytwin boundary configurations and is exposed to external magnetic fields aligned in three directions, is meticulously delineated in Figure 9. The magnetic coercivity, being a crucial parameter, is inherently influenced by the interplay between the specific polytwin boundary types and the orientation of the applied magnetic field. It is noted that, under the imposition of an *x*-axis or *y*-axis magnetic field, the FePt crystals featuring Type I polytwin boundaries exhibit a notably reduced coercive field in comparison with those characterized by Type II boundaries. However, when confronted with a *z*-axis magnetic field, the crystals with Type II polytwin boundaries manifest a discernibly lower coercive field relative to their Type I counterparts. In the context of the mixed polytwin boundary configuration inherent to Type III wherein both Type I and Type II boundaries coexist, the coercive field assumes an intermediary position between the coercive values of Type I and Type II configurations. An important consideration arises from the predominant occurrence of Type I polytwin boundaries over Type II within our simulation of the mixed polytwin boundary (Type III). Consequently, the coercive field associated with Type III aligns more closely with the coercive field exhibited by Type I configurations.

## 4. Conclusions

In summary, the effect of nanostructure on magnetization processes in FePt polytwin crystals was systematically studied by performing phase-field modeling and computer simulations. For FePt polytwin crystals, which are notable for their significant magnetocrystalline anisotropy, the polytwin boundary assumes a pivotal role in influencing magnetization processes in the presence of an external magnetic field. The present investigation leveraged a combination of phase-field modeling and advanced computer simulations to meticulously probe the intricate correlation binding magnetic properties to the microstructural nuances inherent in FePt. In particular, three distinct types of polytwin boundaries have been scrutinized, including symmetric (Type I), asymmetric (Type II), and mixed (Type III) boundary configurations. The primary objective was to comprehensively explore the profound impact of these polytwin boundaries on the magnetization processes as well as the consequential coercive fields experienced under varying external magnetic field orientations. The insightful simulations bring to light the intrinsic dominance of the domain wall motion mechanism in governing the intricate domain switching processes, with the magnetization rotation mechanism only asserting its significance during the advanced stages of magnetization under high external magnetic fields. This phenomenon can be attributed to the substantial magnetocrystalline anisotropy exhibited by the FePt material. Furthermore, among the distinct polytwin boundary configurations investigated, the observed lower coercivity predominantly emanates from the domain wall motion process, which originates from the pivotal intersection point within the polytwin boundary. It is noteworthy that the coercive field pertaining to the mixed polytwin boundary (Type III) consistently assumes an intermediary position between the coercive values exhibited by the Type I and Type II configurations. The consequential findings emanating from this systematic exploration considerably enhance our comprehension of the intricate interplay between polytwin boundaries, magnetization processes, and ensuing magnetic properties within the FePt system.

## Data Availability

Not applicable.
